# Screening of Y chromosome microdeletions in 46,XY partial gonadal dysgenesis and in patients with a 45,X/46,XY karyotype or its variants

**DOI:** 10.1186/1471-2350-14-115

**Published:** 2013-11-05

**Authors:** Ana Paula dos Santos, Juliana Gabriel Ribeiro Andrade, Cristiane Santos Cruz Piveta, Juliana de Paulo, Gil Guerra-Junior, Maricilda Palandi de Mello, Andréa Trevas Maciel-Guerra

**Affiliations:** 1 Department of Medical Genetics, Faculty of Medical Sciences, University of Campinas – UNICAMP, Tessália Vieira de Camargo Street, 126 - Zip Code, 13083-887 Campinas, SP, Brazil; 2 Center of Molecular Biology and Genetic Engineering, University of Campinas – UNICAMP, Campinas, SP, Brazil; 3 Interdisciplinary Group to Study of Sex Determination and Differentiation (GIEDDS), Faculty of Medical Sciences, University of Campinas – UNICAMP, Campinas, SP, Brazil; 4 Department of Pediatrics, Faculty of Medical Sciences, University of Campinas – UNICAMP, Campinas, SP, Brazil

**Keywords:** Partial gonadal dysgenesis, Genital ambiguity, Mosaicism, Microdeletions, Chromosome Y

## Abstract

**Background:**

Partial and mixed gonadal dysgenesis (PGD and MGD) are characterized by genital ambiguity and the finding of either a streak gonad and a dysgenetic testis or two dysgenetic testes. The karyotype in PGD is 46,XY, whereas a 45,X/46,XY mosaicism or its variants (more than two lineages and/or structural abnormalities of the Y chromosome) is generally found in MGD. Such mosaics are also compatible with female phenotype and Turner syndrome, ovotesticular disorder of sex development, and infertility in men with normal external genitalia. During the last few years, evidences of a linkage between Y microdeletions and 45,X mosaicism have been reported. There are also indications that the instability caused by such deletions might be more significant in germ cells. The aim of this work was to investigate the presence of Y chromosome microdeletions in individuals with PGD and in those with 45,X/46,XY mosaicism or its variants and variable phenotypes.

**Methods:**

Our sample comprised 13 individuals with PGD and 15 with mosaicism, most of them with a MGD phenotype (n = 11). Thirty-six sequence tagged sites (STS) spanning the male specific region (MSY) on the Y chromosome (Yp, centromere and Yq) were analyzed by multiplex PCR and some individual reactions.

**Results:**

All STS showed positive amplifications in the PGD group. Conversely, in the group with mosaicism, six individuals with MGD had been identified with Yq microdeletions, two of them without structural abnormalities of the Y chromosome by routine cytogenetic analysis. The deleted STSs were located within AZFb and AZFc (Azoospermia Factor) regions, which harbor several genes responsible for spermatogenesis.

**Conclusions:**

Absence of deletions in individuals with PGD does not confirm the hypothesis that instability of the Y chromosome in the gonads could be one of the causes of such condition. However, deletions identified in the second group indicate that mosaicism may be associated with Y chromosome abnormalities detectable only at the molecular level. If patients with mosaicism and Y microdeletions reared as males decide to undergo *in vitro* fertilization, Y chromosomes which tend to be unstable during cell division may be transmitted to offspring.

## Background

Normal human sex development involves a series of events: sex determination, which is chromosomally established at fertilization (46,XY or 46,XX); differentiation of the gonads into testes or ovaries; and differentiation of internal and external genitalia, which will follow the male pathway in the presence of testicular hormones, or the female pathway in their absence. Factors adversely affecting any stage of these processes can lead to disorders of sex development (DSD), a group of congenital conditions in which the development of the chromosomal, gonadal or anatomical sex is atypical [1].

Partial and mixed gonadal dysgenesis are relatively rare causes of DSD. Individuals with partial gonadal dysgenesis (PGD) are characterized by genital ambiguity due to variable degrees of testicular dysgenesis, absence of syndromic picture and 46,XY karyotype. The histology of dysgenetic testes may vary from gonads with a few tubular structures and predominance of fibrous tissue to those with mild abnormalities, and they may be found bilaterally or associated with streak gonads [2]. The etiology of most cases of PGD is unknown; mutations in *SRY* (*sex determining region Y*) gene are rarely seen [3-5], and in recent years mutations in *NR5A1* (*Nuclear Receptor Subfamily 5, Group A, Member 1*) gene were found in a few patients [6-8].

Mixed gonadal dysgenesis (MGD) is the main differential diagnosis for PGD. Individuals with MGD share similar gonadal and genital features, but the karyotype is 45,X/46,XY or its variants (a 45,X cell line and one or more lineages with a normal or structurally abnormal Y chromosome) [9]. The 45,X/46,XY karyotype may also be found in individuals with female phenotype and Turner syndrome (TS), genital ambiguity and ovotesticular DSD (OT DSD, also known as true hermaphroditism), and in male phenotype with infertility. Whatever the phenotype, this mosaic is associated with short stature, dysmorphisms and cardiovascular and renal anomalies, among other congenital and acquired conditions [10].

Structural rearrangements of the Y chromosome, including deletions, ring chromosomes and isochromosomes may lead to different phenotypes. Yp (short arm) deletions including *SRY* gene directly affect testis differentiation leading to streak gonads and a female phenotype, whereas deletions of the long arm, specially involving the Azoospermia Factor regions AZFa, AZFb and AZFc on Yq11 lead to male infertility [11].

Detection of Y chromosome deletions in routine analysis depends on the size of the missing segment, which may range from a whole chromosome arm, that may be visualized using regular cytogenetic techniques, to a single sequence tagged site (STS) that may only be detected by molecular techniques [12,13]. In recent years, evidences of an association between Y microdeletions and 45,X/46,XY mosaicism have been reported in individuals with TS, MGD and infertility, and there are also indications that the instability caused by such deletions might be more significant in germ cells [12,13].

The aim of this work was to investigate the presence of Y chromosome microdeletions in individuals with 45,X/46,XY mosaicism and its variants bearing different phenotypes and also in patients with PGD, in order to verify if the instability of Y chromosome might be one of the causes of such disorders of gonadal differentiation.

## Methods

### Subjects

Our sample comprised 28 subjects attending a DSD reference service who were divided into two groups, one of patients with PGD (n = 13) and the other of patients with 45,X/46,XY mosaicism or its variants (n = 15).

Inclusion criteria for the group of PGD were 46,XY karyotype; ambiguous genitalia (hypospadias with or without cryptorchidism or typical male genitalia with bilateral cryptorchidism); absence of clinical signs of TS. The presence of one of the following features were also included: a) a streak gonad and a dysgenetic testis; b) two dysgenetic testes; c) a streak gonad or dysgenetic testis and a contralateral testis without evidences of dysgenesis on routine histological analysis or which was not biopsied during surgery due to normal macroscopic appearance; d) hormonal evidence of congenital dysfunction of seminiferous tubules and Sertoli cells (elevated FSH levels detected in the first months of life) when both testes were not biopsied. Data from these patients are shown in Table 1.

**Table 1:** Clinical data from 15 patients with 46,XY partial gonadal dysgenesis

**Case**	**Sex assignment**	**Current age (years)**	**Age at first *visit *** **(months)**	**Urethral meatus**	**Vaginal introitus**	**Right gonad (site,type)**	**Left gonad (site, type)**
1	M	14	0.5	PS	-	LS, DT	LS, S
2	M	20	8	SCR	-	LS, DT	LS, DT
3	F	4	2.5	Normal male	-	AB, DT	I, DT
4	F	16	0.2	SCR	-	AB, DT	AB, S
*5*	M	9	0.7	Norrnal male	-	I,NB^1^	I, NB^1^
6	M	25	4	SCR	-	LS, DT	LS, DT
7	F	19	204	SCR	-	AB, DT	AB, DT
8	M	15	8	Normal male	-	AB, S	AB, DT
9	M	23	1.7	PS	-	LS, NB	I, DT
10	M	2.5	*25*	PS	-	LS, T	AB, S
11	M	17	6	PS	-	I, S	I, DT
12	M	4	6	SCR	-	LS, T	AB, DT
13	M	12	144	PS	-	LS, DT	LS, DT

- absent, + present, *AB* abdominal, *DT* dysgenetic testis, *F* female, *I* inguinal, *LS* labioscrotal, *M* male, *NB* not biopsied, *PER* perineal, *PS* penoscrotal, *S* streak, *SCR* scrotal, *T* testis.

^1^High FSH levels in the first months of life and infancy.

Inclusion criteria for the group with mosaicism were a 45,X/46,XY mosaicism or its variants, regardless of the phenotype. In this group, 11 patients had MGD, one had TS, one had OT DSD, one had typical male genitalia and one had genital ambiguity and palpable gonads in labioscrotal folds, which had not been biopsied, therefore MGD and OT DSD could not be distinguished. Data from these patients are shown in Table 2.

**Table 2:** Clinical data from 15 patients with 45,X/46,XY mosaicism or its variants

**Case**	**Previous karyotype**	**FISH**	**Previous molecular study**	**Phenotype**	**Sex assignment**	**Current age (yrs)**	**Age at first visit (months)**	**Urethral meatus**	**Vaginal introitus**	**Right gonad (site, type)**	**Left gonad (site, type)**
1	45,X/46,XY	-	-	MGD	F	5	1	SCR	-	I, DT	I, DT
2	45,X/46,XY	-	-	MGD	F	23	4	PER	+	LS, T	AB, S
3	45,X/46,XY	-	-	MGD	F	20	3	SCR	-	I, T	I, S
4	45,X/46,XY	-	-	MGD	M	10	21	PEN	-	LS, T	AB, S
5	45,X/46,X,idic(Y)(p11.2)	45,X.ish(DXZ1+, DYZ3-)/46,X,idic(Y).ish idic(Y)(p11.2) (DXZI+, DYZ3++)	-	MGD ? OT DSD?	M	1.5	18	PS	-	LS, NB	LS, NB
6	45,X/46,X,del(Y)(q12)	-	-	MGD	M	19	180	SCR or PS^1^	-	LS, DT	AB, S
7	45,X/46,XY	-	-	MGD	M	1.75	20	normal male	-	AB, NB^2^	AB,NB^2^
8	45,X/46,XY	-	-	MGD	M	7	45	SCR	-	AB, S	LS, T
9	45,X/46,XY	-	-	TS	F	6.5	75	normal female	+	AB, S	AB, S
10	45,X/46,X,+mar	45,X.ish(DXZ1+,DYZ3-)/46,X,+mar.ish der (Y)	Y + ^3^	MGD	F	5	*3*	PS	-	LS, T	AB, S
		(DXZ1+, DYZ3+)									
11	45,X/46,XY	-	-	MGD	M	3	1.5	PS	-	I, S	LS, T
12	45,X/46,XY	-	-	OTDSD	M	0.2	0.1	SCR	-	LS, T	AB, O
13	45,X/46,X,+mar	45,X.ish(DXZ1+,DYZ3-)/46,X,+mar.ish der(Y)	Y + ^3^	MGD	M	17	192	SCR	-	LS,NB^2^	AB, S
		(DXZ1+, DYZ3+)									
14	45,X/46,X,idic(Y)(p11.2)/47,X,idic(Y)(p11.2),idic(Y)(p11.2)	45,X.ish(DXZ1+, DYZ3-)/46,X,idic(Y).ish idic(Y)(p11.2) (DXZ1+,DYZ3++)/		MGD	F	1.5	0.33	PS		LS, T	AB, S
		47,X,idic(Y),idic(Y).ish idic(Y)(p11.2)(DXZ1+, DYZ3++), idic(Y)(p11.2)(DXZ1+,DYZ3++)									
15	45,X/46,X,+mar1/46,X,+mar2/ 47,X,+2mar1/47,X,+mar1,+mar2	45,X.ish(DXZ1+,DYZ3-)/46,X,+mar1.ish dic der(Y)	Y + ^3^	MGD	F	0.2	1	SCR	-	LS, T	I, S
		(DXZ1+, DYZ3++)/									
		46,X,+mar2.ish der(Y)									
		(DXZ1+, DYZ3+)/									
		47,X,+mar1. ish dic der(Y)									
		(DXZ1+, DYZ3++),+mar2.ish der(Y)(DYZ3x1)									

*AB* abdominal, *del* deletion, *DT* dysgenetic testis, *F* female, *i* isochromosome, *I* inguinal, *M* male, *mar* marker, *MGD* mixed gonadal dysgenesis, *NB* not biopsied, *O* ovary, *OT DSD* ovotesticular disorder of sex development, *PEN* penile, *PER* perineal, *PS* penoscrotal, *S* streak, *SCR* scrotal, *LS* labioscrotal, *T* testis, *TS* Turner syndrome. ^1^surgery performed before the first visit; ^2^normal testicular macroscopic appearence on surgery; ^3^positive Y chromosome sequences.

In three patients with mosaicism (cases 10, 13 and 15) karyotype initially revealed a marker chromosome, whose origin was elucidated by the presence of Y-specific DNA sequences (*SRY* and *TSPY* genes and the Y-centromeric region DYZ3) and FISH with DXZ1 and DYZ3 probes (Cytocell Ltd., Cambridge, UK). FISH analysis confirmed the presence of the Y centromere in these cases and determined whether the Y chromosome was monocentric or dicentric. Two other patients (cases 6 and 14) had isodicentric Y chromosomes; in these cases, the presence of two centromeres was observed only in FISH analysis.

This study was approved by local Ethics Committee (Comitê de Ética em Pesquisa - Faculdade de Ciências Médicas - UNICAMP) (1065/2010) and informed consent was obtained from all adult **s**ubjects and from the parents/guardians of all child subjects.

### Molecular study

The screening methods used to identify microdeletions were conventional and multiplex polymerase chain reaction (PCR). Genomic DNA was isolated by Proteinase K (Boehringer Mannheim, Germany) lysis and phenol-chloroform extraction.

We investigated 36 STS located in the Y-specific euchromatic region (short arm - Yp, centromere and long arm - Yq), described in Table 3. In total, 31 primers pairs were distributed in seven different reaction mixes. Five STSs that did not show amplification within mixes prepared for multiplex PCRs were tested separately: DYZ3, TSPY, Y6H35pr, and Y6HPc54pr Y6HP52pr.

**Table 3:** Order and location of STS along the Y chromosome, sequence of the primers used and expected size of the amplified product

**STS**	**Locus**	**Location**	**Chromosomic region**	**Primer *sense***	**Primer *antisense***	Lenght of the amplification product (Bp*)
sY14	SRY	2715341-2715810	p11.31	GAATATTCCCGCTCTCCGGA	GCTGGTGCTCCATTCTTGAG	472
sY1301	BV703591	2881785-2882279	p11.31	GTCTTGTTGCAGCCCATGT	CAAAGGGAGAATAGCAGGC	495
Amely-3	BV678972	6737830-6738427	p11.2	GGTGGGAGAAGGATGTTGTT	AGTCAATCCGAATGGTCAGG	598
sY3218	BV704083	7234129-7234319	p11.2	CTTTTTCGTTGGAGTCTCGC	GCAAAACCCCGTCTCTACAA	191
sY1059	G66106	7561835 – 7562133	q11.223	GATCATTCTCAAGGGGCTCA	TTGTTGTTGTTGTCATTGTGG	299
TSPY	NG022996	9236076-9307357	p11.2	CGATAGGCCTCCACTTCATA	GATGACATAATGGCGGAG	1300
DYZ3	-	-	Centromérica	ACACATCACAAAGAACTATG	TGAAAACTACACAGNAAGCTG	1100
sY81	DYS271	12606410-12606618	q11.1	AGGCACTGGTCAGAATGAAG	AATGGAAAATACAGCTCCCC	209
sY82	DYS272	12838080-12838343	q11.1	ATCCTGCCCTTCTGAATCTC	CAGTGTCCACTGATGGATGA	264
Y6HP35pr	DYS274	ND	-	GGTACACACTCCATCCTGGAC	CTACAGGCTACCTTTTAGGTGG	226
sY86	DYS148	13117503-13117820	q11.1	GTGACACACAGACTATGCTTC	ACACACAGAGCGACAACCCT	320
sY84	DYS273	13299428-13299753	q11.1	AGAAGGGTCTGAAAGCAGGT	GCCTACTACCTGGAGGCTTC	326
sY609	G65840	13488371-13488514	q11.21	CATAGCCCAGAGCAATCCAT	TAGGCATAGGAAGTGGCTGG	144
sY88	DYS276	14113358-14113480	q11.21	TTGTAATCCAAATACATGGGC	CACCCAGCCATTTGTTTTAC	123
sY94	DYS279	14790821-14790970	q11.21	TCATGACAGCCAGGGTATTT	TTTGGACATAGTTTTTTGGTCC	150
sY95	DYS280	15053332-15053634	q11.21	TCCTACAGATGTCCAAAGTGC	GATGAGTGACCCCAGAATTG	303
sY182	KAL-Y	15980790-15980914	q11.221	TCAGAAGTGAAACCCTGTATG	GCATGTGACTCAAAGTATAAGC	125
sY97	DYS281	16263633-16263736	q11.221	AACTTCATCAGTGTTACATCAAGG	TGTGGCATTTTGTTATGTGG	104
sY151	KAL-Y	16020619-16020801	q11.221	AAATCTGTAGTCTCATATCAATCTG	TTACTTGATTTAGCAATAAAAAGG	183
sY102	DYS198	17080238-17080455	q11.221	CACTACCACATTTCTGGTTGG	CGCTGAGTCCATTCTTTGAG	218
sY105	DYS201	17866681-17866981	q11.221	AAGGGCTTCTTCTCTTGCTT	AGGGAGCTTAAACTCACCGT	301
Y6D14pr	DYS205	ND	-	GGCTAGGTGCCAGCAAGTAGATCA	GTTCTCTTCCCCTGCATCAAG	134
sY117	DS209	19171254-19171515	q11.221	GTTGGTTCCATGCTCCATAC	CAGGGAGAGAGCCTTTTACC	262
Y6PHc54pr	ND	ND	-	GCAGAAGAGTTCAGC	GTGGAGTGCTGCATTAAAGG	166
sY1287	G75509	19491288-19491607	q11.221	GCAACATAGATGGACCCAGAA	ATAGCAAAGAGCCTCCCAGA	320
sY1752	BV682283	20229909-20230117	q11.222	TCAGTGTGTCACCAAGCAGG	GAGGCAAGTGGACCATCTGT	209
sY127	DYS218	20979804-20980078	q11.222	GGCTCACAAACGAAAAGAAA	CTGCAGGCAGTAATAAGGGA	274
sY134	DYS224	21965448-21965750	q11.222	GTCTGCCTCACCATAAAACG	ACCACTGCCAAAACTTTCAAA	301
sY143	DYS231	22387291-22387601	q11.223	GCAGGATGAGAAGCAGGTAG	CCGTGTGCTGGAGACTAATC	311
sY1059	G66106	22822646-22822944; 23252550-23252848	q11.223	GATCATTCTCAAGGGGCTCA	TTGTTGTTGTTGTCATTGTGG	299
sY149	DYS1	23725053-23725184	q11.223	TGTCACACTGCCCTAATCCT	TGGTCATGACAAAAGACGAA	132
sY147	DYS232	23882766-23882865	q11.223	TTTCTCGTTTGATGATCCTAG	TTAATATGAGAATGAGAACAGATGT	100
Fr15-Hpr	ND	ND	-	TACCTTGGTTTTGCACCAGACGC	CACCCTCTGTATATGACCTGGC	313
Y6HP52pr	DYS239	ND	-	GGAACTGGCAGGATTAGCTTC	GCTCAGAATCTGCGATCAG	258
sY1059	G66106	24054410-24054708	q11.223	GATCATTCTCAAGGGGCTCA	TTGTTGTTGTTGTCATTGTGG	299
sY157	DYS240	24284845-24285134	q11.223	CTTAGGAAAAAGTGAAGCCG	CCTGCTGTCAGCAAGATACA	285
sY1191	G73809	24875620-24876004	q11.23	CCAGACGTTCTACCCTTTCG	GAGCCGAGATCCAGTTACCA	385
sY1059	G66106	26726968-26727266	q11.223	GATCATTCTCAAGGGGCTCA	TTGTTGTTGTTGTCATTGTGG	299
sY3168	BV704033	28761338-28761820	q11.23	GCACCACTGTACTGAAGCATG	TCCCAGCTCACATTGATGATTA	483

*Bp basepairs.

Multiplex PCR was carried out in a total volume of 26 μL containing 10× PCR buffer, 1.5 mM MgCl2, 1.25 mM dNTPs (deoxyribonucleotide Tri-Phosphate), DMSO (Dimethylsulfoxide) and 0.5 U Taq (Thermus aquaticus) DNA polymerase (Invitrogen, USA) with 200 to 500 ng of genomic DNA. DNAs from males with 46,XY normal karyotype and normal genitalia were used as positive control and the genomic DNAs from females with normal karyotype and genitalia were used as the negative control in each reaction.

The samples were subjected to PCR amplification using initial denaturation for 5 minutes at 94°C, and 35 cycles of 94°C for 30 seconds, annealing step at 58°C for 45 seconds and extension step at 62°C for 2 minute. The final extension time was 5 minutes at 65°C. Conditions for PCR were the same for all seven mixes. We used the GeneAmp PCR System 9700 thermocycler - Applied Biosystem.

Conventional PCRs were used to test five STSs individually and also to confirm apparent deletions in multiplex PCR, discarding the possibility that the absence of amplification was due to some artifact in the multiplex PCR reaction. The PCR was standardized in a reaction with a final volume of 25 μL. Negative and positive reaction controls were the same as described above.

Gel electrophoresis with 3% agarose in 1 × TBE buffer was used for multiplex PCR products; whereas 1% agarose gel was used for conventional PCR products.

An STS was considered absent only after at least two amplification failures in the presence of positive control. To confirm these deletions, two more individual PCRs were performed.

## Results

**Figure 1:**
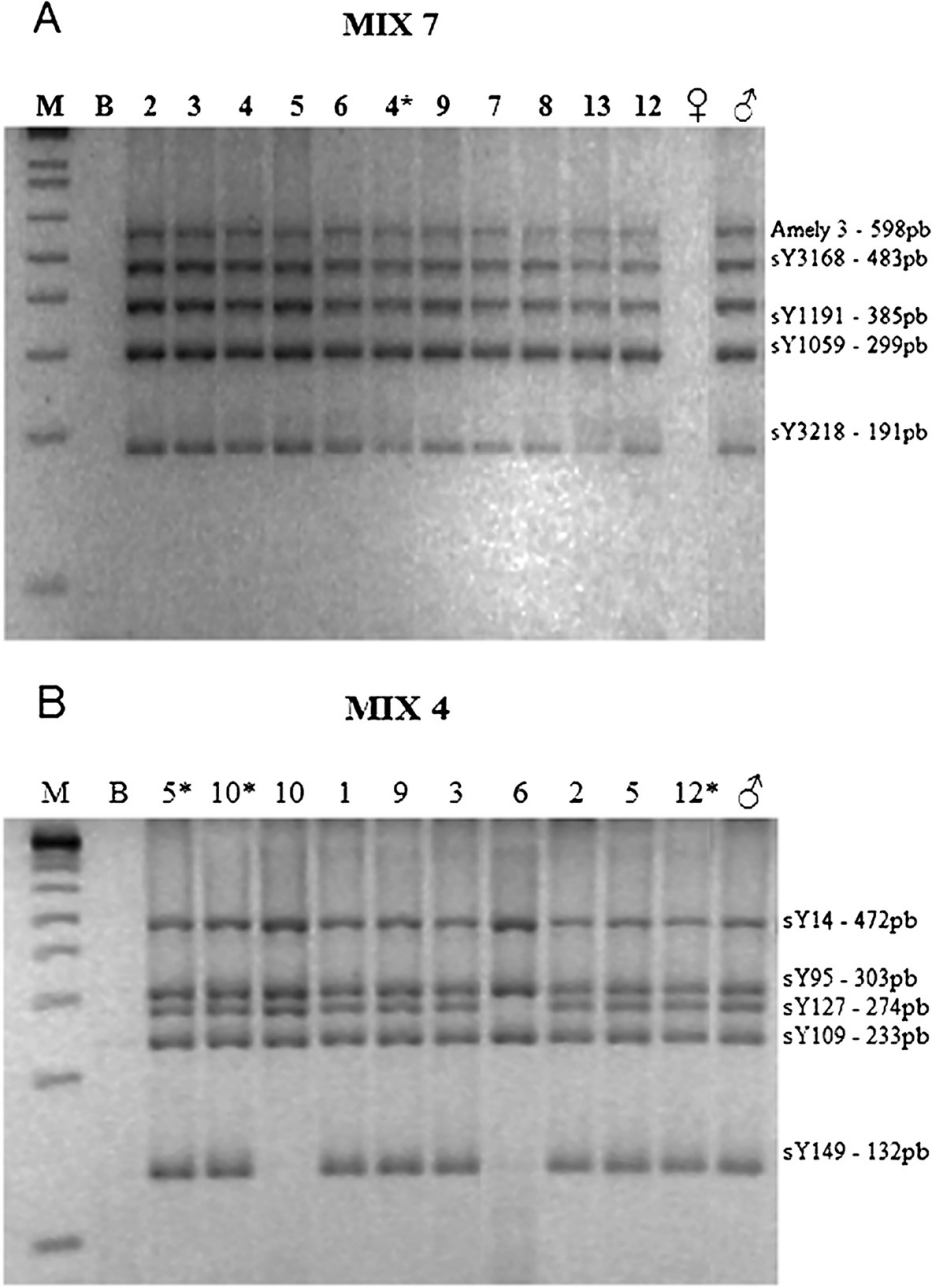
**Agarose gel stained with ethidium bromide showing the PCR fragments of MIX 7 and 4. A** – Five STS were amplification in all patients of DGP group. M – Molecular weight marker (Ladder 1Kb); B – Blank; Individuals: 2, 3, 4, 5, 6, 9, 7, 8, 13 e 12 (Table 1) e 4* (Table 2); ♀ – Female control; ♂ - Male control. **B** – Absent amplification of STS sY149 for individuals 10 and 6, and no amplification of STS sY127 for individual 6. M – Molecular weight marker (Ladder 1Kb); B - Blank; Individuals 5*, 10*, 12* (Table 1) and individuals 10, 1, 9, 3, 6, 2, 5 (Table 2); ♀ – Female control; ♂ - Male control.

All patients with PGD had positive amplification for every STS, indicating absence of deletions. Figure 1 illustrates the absence of microdeletions in one of the mixes from patients with PGD.

**Figure 2:**
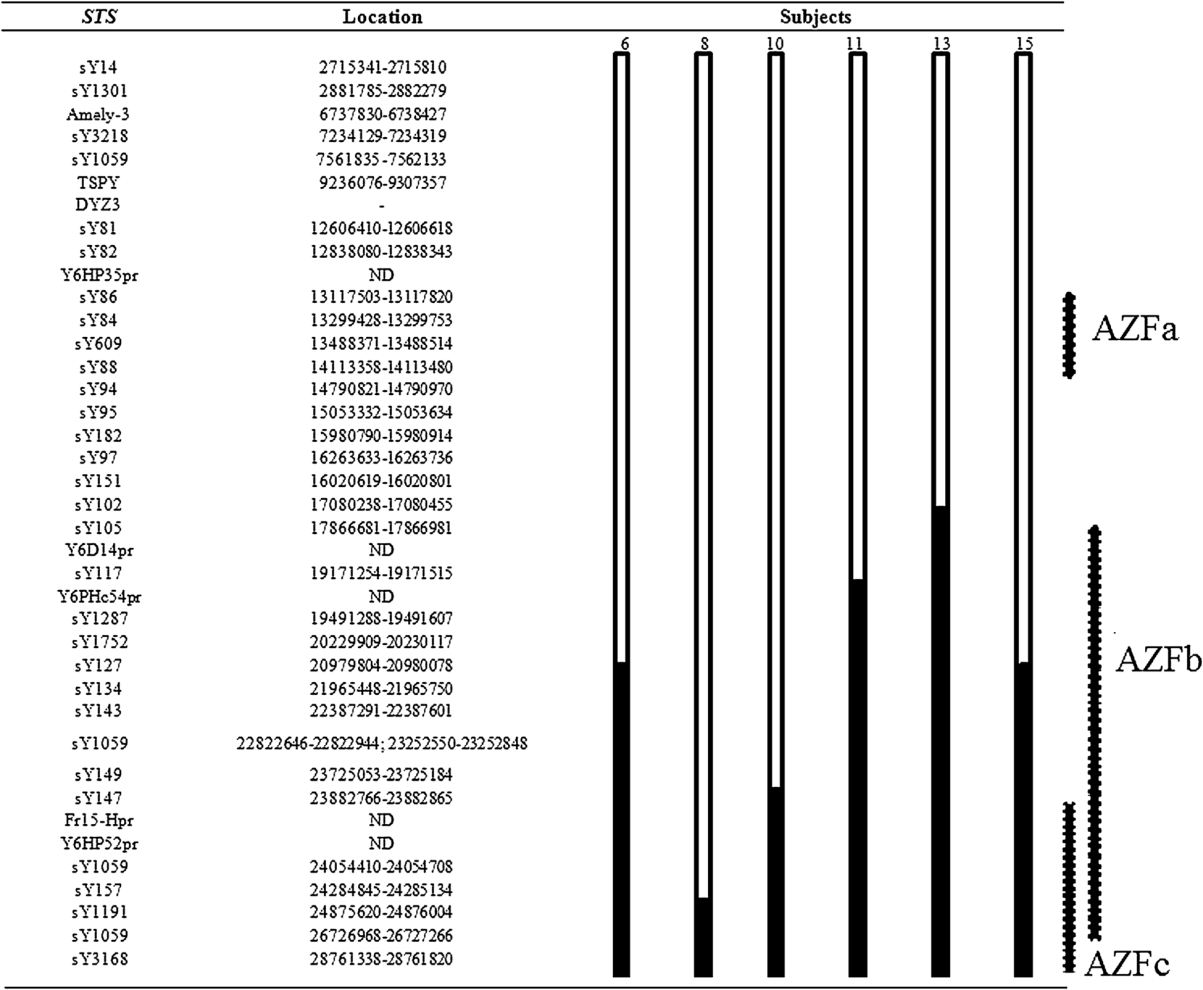
**Schematic description of microdeletions found in six individuals in the MOS group.** The figure shows the location of identified deletions for each patient and the position of each deletion in relation to the AZFa, AZFb and AZFc regions.  = Fragment present.  = Fragment absent.

In the group of patients with mosaicism, six out of 15 individuals showed absence of amplification of some STS indicating the presence of microdeletions. Two of them had a 45,X/46,XY karyotype, while in the other four an abnormal Y chromosome had been detected in cytogenetic analysis. All microdeletions were located in the long arm, with different breakpoints. Figure 1 shows some of these findings. Confirmation of microdeletions detected by multiplex PCR was achieved by two individual PCRs of each STS. Figure 2 shows the location of the identified deletions for each patient and their position in relation to AZF regions. Two of the nine patients without deletions in the male-specific region on the Y chromosome (MSY) had isodicentric Y chromosomes.

## Discussion

The Y chromosome evolved from an autosome, and its evolution has been characterized by massive gene decay, the human MSY retains only three percent of the ancestral autosome genes [14,15]. Human Y chromosome has a total length of approximately 60 MB and is composed of two pseudoautosomal regions (PAR1 and PAR2), the euchromatic region, which is rich in testis-specific genes, and the heterochromatic region, containing highly repetitive satellite DNA. The euchromatic region and some of the heterochromatic parts of the Y chromosome are called MSY (male-specific region on the Y chromosome), which comprises 95% of the chromosome’s length [16,17]. MSY contains 496 genes, including 51 coding genes, 326 pseudogenes, and 119 non-coding genes (Ensembl Genome Browser version 69, accessed January 2013) [18].

The present study did not detect Y chromosome microdeletions in the 13 subjects with PGD and normal 46,XY karyotype, therefore the hypothesis that abnormalities at the molecular level leading to loss of the Y chromosome in gonadal cell lines play a role in the etiology of this disorder was not supported. To the best of our knowledge, this is the first study to investigate the association between Y chromosome abnormalities and PGD. However, the hypothesis that mosaicism restricted to testis could be one of the causes of this condition could not be entirely discarded, because the loss of the Y chromosome giving rise to a 45,X cell line can occur in the absence of structural abnormalities of the Y chromosome. Furthermore, microdeletions found on gonadal DNA can be absent on peripheral blood lymphocyte DNA [19].

On the other hand, microdeletions were found in six of 15 patients with a 45,X/46,XY karyotype or its variants, all of them with MGD phenotype. Proximal breakpoints inside the AZF region were defined in four of the six carriers of structural Y chromosome abnormalities which had already been detected in routine karyotype. The molecular study of Y-STS not only allowed to refine the breakpoints, but also made it possible to detect the loss of euchromatic regions of this chromosome.

In addition, in two of nine individuals with an apparently normal Y chromosome (cases 8 and 11) the abnormalities were detected only by the molecular techniques. This frequency (2/9) was similar to that observed by Alvarez-Nava *et al.* (2008) (3/11) [19] and lower than that found by Patsalis *et al.* (2005) (4/7) [12]. Taken together, these findings suggest that 9/27 or one in three individuals with 45,X/46,XY and apparently normal Y chromosome may have Y microdeletions.

All alterations identified in the six individuals were located on the Y chromosome long arm at the beginning of Yq11.221 region, where the AZFb region is located, and extending to AZFc. Only in case 8 the deletion was practically limited to the AZFc region. In the four cases in which an abnormal Y chromosome had already been detected, Y-STS testing were not useful to define distal breakpoints, which might be located between the q11-q12 boundary and terminal Yq. In case 11 the analysis of Y-STS showed that the lost fragment was not confined to the heterochromatic region.

The same fragment in the euchromatic region was lost in cases 6 and 15; the deletion starts at STS sY127, located in Yq11.222, and includes 11 STS. In the karyotype of case 6, only a Yq12 deletion was detectable, while in case 15 there were four cell lines containing markers with different Yq sizes. In case 8, two STS were missing (sY157 and sY3168), indicating a breakpoint within Yq11.223. Upon karyotyping, there was an apparently normal Y chromosome. The deletion in case 10 started at sY149 (Yq11.223), with absence of seven STS.

Deletions found in cases 11 and 13 led to loss of 13 and 17 STS, respectively. In patient 11, whose karyotype was 45,X/46,XY, the missing fragment started in sY117 (Yq11.221) and included all subsequent STS. In case 13 the deletion started at STS sY105, also located in Yq11.221; the karyotype had already detected a structural chromosome abnormality with loss of the heterochromatic region Yq12.

In cases 10, 13 and 15, in which there were Y-derived markers, STS study detected the presence of a terminal deletion of the euchromatic region; deletion in case 10 was the smallest, encompassing AZFc and the overlapping region of AZFb and AZFc.

No deletions (including those of Yp) were found in the two subjects with isodicentric Y chromosomes (cases 5 and 14); these chromosomes are often unstable during cell division, resulting in mosaicism with a 45,X line [20]. As the STS analyzed in this study included all the euchromatic region of Yp from the centromere to p11.31, the break in the short arm that originated these isodicentrics may have occurred in the PAR1 region. It has been suggested that a hot spot located in the PAR1 is prone to chromosomal breakage and reunion with generation of isodicentric Y chromosomes [20].

Yq microdeletions with loss of genes specifically expressed in the testis directly contribute to male infertility. AZFc is most often involved, followed by AZFb, and more rarely AZFa [21-23]. In addition, deletions involving the entire AZFb region or AZFb-c remove a large stretch of Yq chromatin, which may result in more severe disturbance of X-Y chromosome pairing during meiosis than isolated AZFc deletion leading to meiotic breakdown [24,25]. All microdeletions found in our patients with mosaicism were located in AZFb and AZFc regions; their effect on Y chromosome instability is not mediated by the loss of function of genes related to spermatogenesis, but one could speculate that the loss of these large stretch of Yq could also be more dangerous in mitosis than AZFc deletions alone in increasing the risk of mosaicism due to loss of the abnormal Y chromosome.

The mechanism of Y chromosome deletions is accidental homologous recombination between highly similar or identical sequences, which are found in great abundance on this chromosome [11]. Three of our patients (cases 8, 10 and 11) had a breakpoint within regions consisting of palindromes [26,27], which may have contributed to the emergence of these deletions [26,27].

Most genes involved in Y chromosome deletions are expressed specifically in the testes during spermatogenesis, but do not appear to be essential for fertilization or embryogenesis; thus, these deletions does not seem to adversely affect the fertilization results in men whose sperm was obtained by testicular sperm extraction (TESE). Thus, subjects with MGD may also benefit from procedures such as TESE followed by *in vitro* fertilization with intracytoplasmic sperm injection (ICSI) [11].

Thereby, patients with a 45,X/46,XY karyotype or its variants reared as males who have Y microdeletions may, by means of assisted reproductive technologies, generate not only male offspring with sterility, but also individuals who are carriers of an unstable Y chromosome that may originate mosaicism with a 45,X cell line in the early stages of embryonic development leading to various anomalous phenotypes (TS, MGD and OT DSD) [13]. This is the case of the three patients with microdeletions from this sample reared as males, two with an apparently normal Y chromosome (cases 8 and 11) and one with a deletion apparently limited to Yq12 (case 6). In these three cases, as well as in case 13, in whom the structural alteration of the Y was more evident on karyotype, the presence of these abnormalities in euchromatic region should be taken into account in counseling regarding assisted reproduction.

## Conclusions

Absence of deletions in individuals with PGD suggests that the Y chromosome is intact, indicating that it is not likely that its instability in the gonads is one of the causes of this condition; further studies that aim to identify the etiology of this condition are needed. However, deletions identified in the second group indicate that mosaicism may be associated with Y chromosome abnormalities detectable only at the molecular level. If patients with mosaicism and Y microdeletions reared as males decide to undergo *in vitro* fertilization, Y chromosomes which tend to be unstable during cell division may be transmitted to offspring.

## Abbreviations

DSD: Disorders of sex development; PGD: Partial gonadal dysgenesis; MGD: Mixed gonadal dysgenesis; SRY: Sex determining region Y; NR5A1: Nuclear Receptor Subfamily 5, Group A, Member 1; TS: Turner syndrome; OT DSD: Ovotesticular disorders of sex development; Yp: Short arm of Y chromosome; Yq: Long arm of Y chromosome; AZF: Azoospermia factor region; STS: Sequence tagged sites; FSH: Follicle stimulating hormone; dNTP: (Deoxyribonucleotide Tri-Phosphate); DMSO: Dimethylsulfoxide; Taq: Thermus aquaticus; DNA: Deoxyribonucleic acid; TBE: Tris-borate EDTA; PAR1: Pseudoautosomal regions; TESE: Testicular sperm extraction; ICSI: Intracytoplasmic sperm injection.

## Competing interests

The authors declare that they have no competing interests.

## Authors' contributions

APS participated in the design of the study, carried out the molecular genetic studies and drafted the manuscript. JGRA participated in the design of the study, contacted the patients and provided clinical data. CSCP carried out the molecular genetic studies. JP participated in the design of the study and provided cytogenetic data. GGJr participated in the design of the study and provided clinical data. MPM conceived of the study, participated in its design and coordination and helped to draft the manuscript. ATMG conceived of the study, participated in its design and coordination and helped to draft the manuscript. All authors read and approved the final manuscript.

## Pre-publication history

The pre-publication history for this paper can be accessed here:

http://www.biomedcentral.com/1471-2350/14/115/prepub
